# Amantadine for functional improvement in patients with traumatic brain injury: A systematic review with meta-analysis and trial sequential analysis

**DOI:** 10.1016/j.bas.2024.102773

**Published:** 2024-02-23

**Authors:** Hantz Filbert C. Siy, Michael Louis A. Gimenez

**Affiliations:** University of Santo Tomas Hospital, España Blvd., Sampaloc, Manila, Philippines

**Keywords:** Amantadine, Traumatic brain injury, Trauma, Brain injury, Functional outcome

## Abstract

**Introduction:**

TBIs contribute in over one-third of injury-related deaths with mortality rates as high as 50% in trauma centers serving the most severe TBI. The effect of TBI on mortality is about 10% across all ages. Amantadine hydrochloride is one of the most commonly prescribed medications for patients undergoing inpatient neurorehabilitation who have disorders of consciousness.^6^ It is a dopamine (DA) receptor agonist and a N-Methyl-D-aspartate (NMDA) receptor antagonist via dopamine release and dopamine reuptake inhibition. The current study will synthesize the current available evidence and show the effect of Amantadine in functional improvement after TBI.

**Research question:**

Does Amantadine have an effect on functional improvement of TBI patients?

**Material and methods:**

This systematic review included all randomized placebo-controlled trials that compare the use of Amantadine versus placebo for functional improvement of patients after TBI. Outcome measures included DRS, GCS and/or GOS scores.

**Results:**

Three studies with a total of 281 patients were included in the quantitative analyses. GRADE assessments show that there was a high certainty of evidence for functional improvement in terms of DRS scores.

**Discussion and conclusion:**

Evidence of this review show that the use of Amantadine may have a beneficial effect on functional outcome in moderate to severe traumatic brain injuries among adult patients. Given the still-limited body of knowledge, more relevant studies must be made exploring the impact of Amantadine therapies on promoting functional recovery within the brain injury rehabilitation care continuum, with the goals of achieving larger sample sizes and establishing the early- or later-treatment beneficial effects.

## Highlights

• Amantadine hydrochloride is a dopamine (DA) receptor agonist and a N-Methyl-D-aspartate (NMDA) receptor antagonist via dopamine release and dopamine reuptake inhibition.
• Amantadine versus placebo for functional improvement of patients after TBI. Outcome measures included DRS, GCS and/or GOS scores.
• GRADE assessments show that there was a high certainty of evidence for functional improvement in terms of DRS scores, mainly because the effect estimates derived from the pooled studies possess low to unclear risks of bias, are directly applicable to intended trauma patients and could potentially demonstrate dose-gradient effects as implied on the Amantadine regimen employed in the studies.
• Evidence of this review show that the use of Amantadine may have a beneficial effect on functional outcome in moderate to severe traumatic brain injuries among adult patients.

## 1 Introduction

### 1.1 Description of the condition

According to the US Centers for Disease Control and Prevention (CDC), traumatic brain injury (TBI), which can range in severity from mild to severe, is brought on by a penetrating head injury as well as by a bump, blow, or jolt to the head. The severity of the signs and symptoms varies, ranging from brief loss of consciousness (LOC) to seizures, coma, or even death (Faul and Coronado, 2015).

Glasgow Coma Scale (GCS) score is one of the most popular ways to assess the severity of TBI, and is frequently used in prehospital setting and in the emergency department (ED). The individual elements each reveal the patient's crude functional status, and collectively is demonstrated to be a reliable approach for estimating the severity of TBI (Faul and Coronado, 2015). Another assessment tool called Glasgow Outcome Scale (GOS), which assesses disability and social involvement, is also the most used outcome measure in studies on brain damage. The GOS has been employed in more than 90% of the most robust trials and is the most common clinician-reported outcome assessment for randomized clinical trials in TBI (McMillan et al., 2016). Secondarily, the Disability Rating Scale (DRS) is also employed among TBI patients. The scale ranges from 0 (no disability) to 29 (extreme vegetative state). Although relatively short, it is more thorough than other scales since it incorporates items that assess Impairment, Disability, and Handicap—three categories specified by the World Health Organization (WHO). It is more sensitive than the GCS and GOS in measuring clinical changes in severe TBI patients and inpatient rehabilitation. This scale's limitation is that it is unable to detect subtle changes that may appear in higher functioning people, especially moderate TBI survivors (Le Roux et al., 2013).

TBIs contribute in over one-third of injury-related deaths with mortality rates as high as 50% in trauma centers serving the most severe TBI (Faul et al., 2002–2006). Younger and middle-aged groups bear the burden of injury, with 15% disability-adjusted life years lost because of injury. The effect of injuries on mortality is about 10% across all ages. More specifically, from 2002 to 2006, there were 1.7 million TBI cases annually in the US alone (579 per 100,000 people) (Faul and Coronado, 2015). A TBI also may result in disability and death even after one year of hospitalization: good recovery, 32%; moderate disability, 14%; severe disability, 24%; vegetative status, 1%; and death, 29% (Jiang et al., 2002).

### 1.2 Description of the intervention

Amantadine hydrochloride (also, Amantadine) is one of the most commonly prescribed medications for patients undergoing inpatient neurorehabilitation who have disorders of consciousness (Whyte et al., 2005). It is a dopamine (DA) receptor agonist and a N-Methyl-D-aspartate (NMDA) receptor antagonist via dopamine release and dopamine reuptake inhibition (Sawyer et al., 2008).

### 1.3 How the intervention might work

Its mechanism of action appears to support the current hypothesis that TBI cognitive deficits are caused by disruptions in the dopaminergic and glutamatergic pathways (Kornhuber and Weller, 1997). As a DA receptor agonist, it helps stimulate recovery of nervous system after TBI. As a direct antagonist of NMDA-receptor and down-regulator of glutaminergic pathways, Amantadine inhibits activity of post-synaptic membrane calcium channels consequently reducing the uptake of calcium into the neurons. This results in neuroprotective effects in severe TBI even after months of follow-up (Bullock, 1993; Gualtieri et al., 1989).

### 1.4 Why it is important to do this review

Comparisons of TBI outcomes are challenging due to methodological differences and variations in healthcare systems and clinical trial designs. The majority of the differences were caused by broader TBI admission criteria based on different case definitions and patient inclusion rules, which pose a challenge in improving clinical outcomes of TBI patients, particularly in moderate to severe cases. Furthermore, neuropharmacological therapies are often used off-label to improve responsiveness, on the premise that supplementation can improve injury-induced impairment in dopaminergic and noradrenergic neurotransmitter systems. However, despite promising results, data are opposing questioning the topic anew. Thus, the current study will synthesize the current available evidence and show the effect of Amantadine in functional improvement after TBI.

### 1.5 Research question

Does Amantadine have an effect on functional improvement of TBI patients?

### 1.6 General objective

The objective of this review is to determine the effect of Amantadine on functional improvement as compared to placebo among adult TBI patients.

### 1.7 Specific objective

Specifically, this study aims to.

1. To describe the characteristics of the studies included in this review.
2. To determine the effect of Amantadine on functional improvement after TBI.

## 2 Methods

### 2.1 Review protocol

The conduct and reporting of this review followed the up-to-date version of the Methodological Expectations of Cochrane Intervention Reviews (MECIR) manual, compatible with the core items of the Preferred Reporting Items for Systemic Review and Meta-analysis (PRISMA) 2020 Checklist, as recommend by the Cochrane Handbook for Systematic Reviews of Interventions.

### 2.2 Criteria for considering studies for this review

This systematic review included all randomized placebo-controlled trials that compare the use of Amantadine versus placebo for functional improvement of patients after TBI. Studies published in any language was included. Participants included all patients with TBI. The comparator groups were Amantadine versus placebo.

### 2.3 Types of outcome measures

#### 2.3.1 Primary outcome

• Outcome prioritization: functional improvement as measured using DRS.
• Exploratory outcome: Mean GCS or GOS scores if any were reported and if meta-analysis were possible.

### 2.4 Exclusion criteria

Qualitative studies, reviews, case series/reports, animal studies, letters and editorials were excluded.

### 2.5 Search methods for identification of studies

A systematic search of electronic medical literature databases including Cochrane Library, PubMed, Embase and clinicaltrials.gov was conducted from inception until September 2022. Manual search of the reference lists of the retrieved articles was done. An electronic literature search was done using subject headings and keywords related to the terms including “Amantadine” and “traumatic brain injury” with key-term sequence ((“amantadine”) OR (amantadine*)) AND (((“Severe Traumatic Brain Injury”) OR (Traumatic Brain Injury*)) OR (((severe) AND ((traumatic*) OR (trauma))) AND ((“brain injury”) OR (brain injury*)))).

### 2.6 Study selection

Eligibility of studies for inclusion that was obtained from the electronic and manual searches were assessed. The titles and abstracts were evaluated for preliminary screening. Studies that meet the inclusion criteria were included in the second screening. The full-text articles of these studies were retrieved and analyzed in the second screening. In the event of disagreement, an independent reviewer was consulted.

### 2.7 Data extraction and management

The following data from each included study were extracted: (1) author, (2) year of publication, (3) setting, (4) sample size, (5) age of participants, (6) severity of brain injury, (7) intervention used (Amantadine versus placebo), (8) duration of administration or follow-up, (9), (10) reported DRS, and (12) study design. In case of disagreement, an independent reviewer was consulted.

### 2.8 Assessment of risk of bias

Full-text studies were appraised using the Cochrane risk of bias version 2 (ROB-2) tool (Higgins et al., 2022a). The assessment included randomization, blinding of participants and personnel, blinding of outcome assessment, deviation from intended intervention, missing outcome data, measurement of the outcome, and selection of the reported result. Risk of bias summary and graph were generated using RevMan 5.4.1. In the event of disagreement, an independent reviewer was consulted.

### 2.9 Data analysis

In the presence of statistical, methodological and clinical heterogeneities across the included studies in a meta-analysis, both fixed-effect and random-effects models were reported as a pragmatic and practical approach recommended by Higgins et al. (Deeks et al., 2022) To indicate a statistically significant difference, the generated P-values were two-sided and a P < 0.05 was considered (Higgins et al., 2022b). Interstudy variations and statistical heterogeneities will be estimated using Cochran's Q-statistic with P-values <0.05 indicating statistically significant heterogeneities. The present study will also quantify the effect of heterogeneity by using the i^2^ index (range, 0–100%), which represents the proportion of interstudy variability attributed to heterogeneity, rather than to chance (Higgins et al., 2022b).

Standardized mean difference (SMD) was used as an effect measure in the primary meta-analysis when data were reported in different scales or units of expression to measure the size of the intervention effect relative to the between-participant variability in functional improvement with a value of 0 representing no difference or null effect between the Amantadine and placebo groups.^17 13^ Outcome prioritization focused on DRS to study the influence of Amantadine on functional improvement (Le Roux et al., 2013). The statistical significance of the pooled effect was examined by the Z-test (Higgins et al., 2022b). As this review focused on the long-term effects of Amantadine, cross-over studies in which patients who were switched to both Amantadine and placebo at different timepoints were not included.

Since the amount of the available evidence using conventional meta-analytic methods could commonly take for granted the reliability of statistically significant effects, and P-values based on a limited number of events and/or patients were often not reliable, (Higgins et al., 2022b) trial sequential analysis (TSA) was employed. Meta-analytic sample size considerations, adjusted statistical monitoring boundaries, and adjusted repeated significance testing on accumulating data were utilized based on advanced probability theories to account for the strength of the available evidence and to control the risk of type I and type II errors when repeated significance testing occurred (Pogue and Yusuf, 1997, 1998; Thorlund et al., 2009; Wetterslev et al., 2008; Brok et al., 2008, 2009).

The required information size (RIS) in TSA was obtained to detect a realistic intervention effect for a conclusive and reliable meta-analysis (Pogue and Yusuf, 1997, 1998; Thorlund et al., 2009; Wetterslev et al., 2008, 2009; Brok et al., 2008, 2009). Heterogeneity-adjustment factor and anticipated or observed effect size of Amantadine were incorporated in determining the RIS to account for the increase in variation that a meta-analysis incurs (Thorlund et al., 2017). The effect measure used in TSA were expressed as mean difference (MD) to generate the RIS, monitoring boundaries and futility boundaries.

In a meta-analysis with TSA producing monitoring boundaries, adjusted repeated significance testing used series of Z-values in obtaining cumulative Z-scores plotted with respect to the accumulating data across studies (Pogue and Yusuf, 1997, 1998; Thorlund et al., 2009; Wetterslev et al., 2008; Brok et al., 2008, 2009). To minimize exaggeration of type I errors in producing monitoring boundaries, where the risk of random error and time-trend biases were of particular concerns at early stages of each study, the collation of thresholds for the Z-curve was adjusted by employing O'Brien-Fleming α-spending function accounting for the degree of fluctuations due to the random error, heterogeneity and previous significance testing as a result of accumulating data (DeMets and Lan, 1994; Lan and DeMets, 1983).

Testing futility boundaries using β-spending function in meta-analysis were also employed to control type II errors when a result is found to be non-significant and to know whether the findings were due to lack of power or the intervention likely to provide a threshold for no effect or an underlying equivalency between interventions exists (Thorlund et al., 2017). Z-values within the area of equivalence which did not cross either the futility boundaries for non-superiority (upper boundary) or non-inferiority (lower boundary) were considered equivalent (Thorlund et al., 2017). Z-curve which did not cross the boundary for statistical significance (Z = 1.96) and the futility boundaries for non-superiority were considered inconclusive. Meta-analyses that surpassed the boundary for statistical significance (Z = 1.96), and RIS or the O'Brien-Fleming superiority boundaries in favor of the Amantadine should have enough power to demonstrate superiority over the other (Thorlund et al., 2017).

### 2.10 sensitivity analysis

To describe how the completeness of studies impact the conclusion of this review, synthesis was carried out by restricting the primary analyses to full-text studies at low or unclear risk of bias only, and by undertaking sensitivity analyses to ascertain if incomplete reporting of studies and severity of TBI would change the direction of results.

### 2.11 Certainty assessment

Certainty of evidence assessments on the outcome level were employed using the Grading of Recommendations, Assessment, Development and Evaluation (GRADE) Approach Handbook. GRADE profiler (GRADEPRO) was utilized to import data from Review Manager 5.4.1 (Review Manager) and to create ‘Summary of findings' (SoF) tables.

## 3 Results

### 3.1 Description of studies

#### 3.1.1 Results of the search

**Fig. 1:**
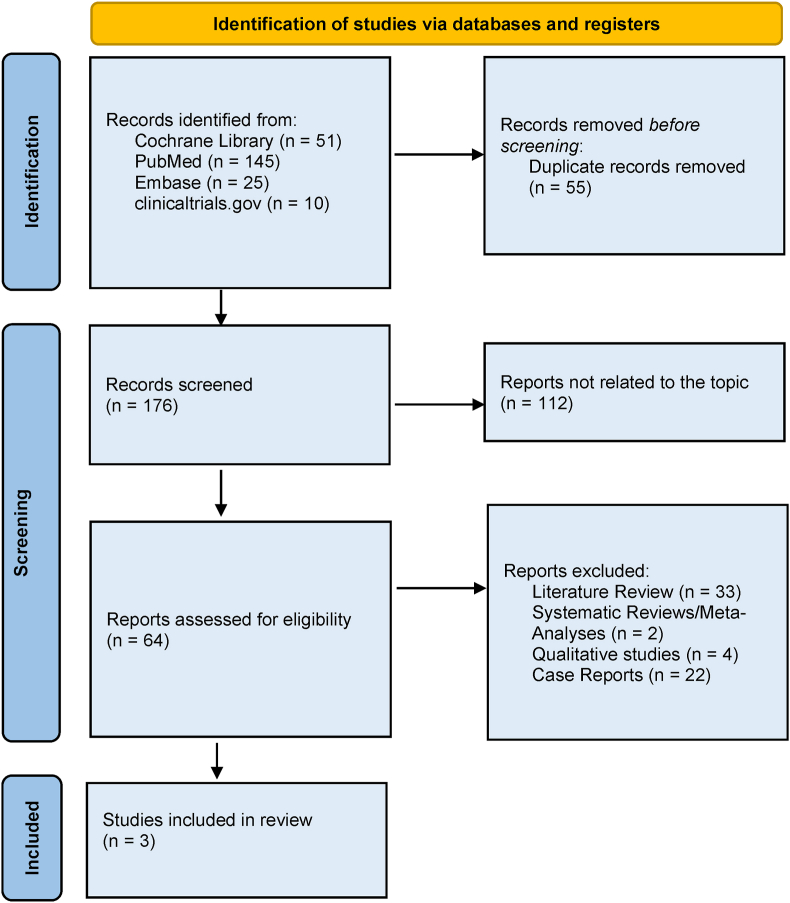
Study flow diagram.

A total of two hundred thirty-one (231) literatures were found after searching Cochrane Library, PubMed, Embase and clinicaltrials.gov from which 55 articles were duplicates and excluded. As outlined in Fig. 1 upon eligibility screening, 64 publications were assessed and 59 publications were excluded (reasons for exclusion: literature reviews, systematic reviews and/or meta-analyses, qualitative studies and case reports/series.

#### 3.1.2 Included studies

Three studies (Ghalaenovi et al., 2018; Giacino et al., 2012; Shimia et al., 2021) with a total of 281 patients (135 patients belonged to Amantadine group and 146 were in the placebo group) were included in the quantitative analyses. The included studies were published between the years 2012–2021. The intervention groups, samples analyzed, study characteristics, and participant baseline features for each outcome were summarized in Table 1.

**Table 1:** Baseline features of the studies included in the analysis.

Study	Study Design/Blinding/Randomization	Patients in Amantadine Group	Patients in Placebo Group	Dosage/Regimen	Severity	Ascertainment Measure	Timepoints	Enrollment Period	Location
Ghalaenovi et al., 2018 (Ghalaenovi et al., 2018)	RCT/Double-blinded/Blocked randomization	19	21	100 mg Amantadine bid for 6 weeks until end of study period	Moderate-severe (GCS lower than 10 on the beginning day)	DRS	6 months	Jan 2013–Apr 2014	Iran
Giacino et al., 2012 (Giacino et al., 2012)	RCT/Double-blinded/Blocked randomization	87	97	100 mg Amantadine bid for 2 weeks; increased to 150 mg Amantadine bid at 3rd week; 200 mg Amantadine bid at 4th week if no improvement from baseline	Severe (vegetative state or a minimally conscious state, DRS score >11)	DRS	4 weeks	–	USA, Denmark, Germany
Shimia et al., 2021 (Shimia et al., 2021)	RCT/Triple-blinded	29	28	100 mg Amantadine bid for 2 weeks; increased to 150 mg Amantadine bid at 3rd week; 200 mg Amantadine bid at 6th week	Severe	DRS (change from baseline)	14–42 days	–	Iran

Reported severity ranged from moderate to severe across studies. Dosage and intervals, timepoints/follow-up, blinding, and locations also posed methodological and clinical heterogeneities in this current study.

#### 3.1.3 Risk of bias in included studies

**Fig. 2:**
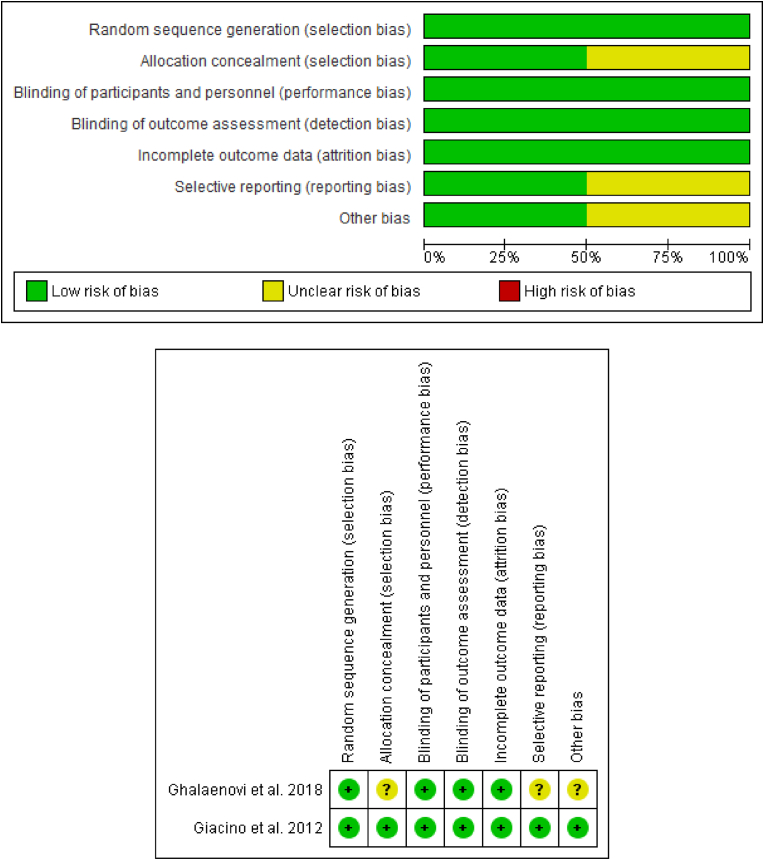
Risk of bias summary.

The assessment for the risk of bias in the included studies is shown in Fig. 2 below.

#### 3.1.4 Effects of interventions

See SoF and GRADE evidence profile tables on Amantadine groups versus placebo groups for the reported functional improvements using DRS among moderate-severe TBI patients in the Supplementary Information section. GRADE assessments on functional improvement suggested high certainty of evidence.

#### 3.1.5 Primary Analysis

**Fig. 3:**
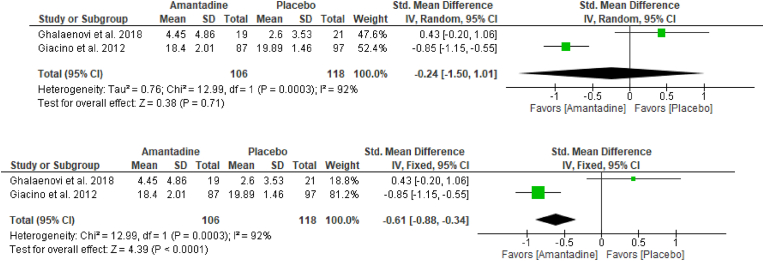
Forrest plot via random-effects model (Fig. 3A, top) and fixed-effect model (Fig. 3B, bottom) of Amantadine versus placebo on functional improvement among moderate-severe TBI patients (Primary Analysis).

Although the trend leaned towards the Amantadine group, random-effects analysis showed no significant difference [SMD: -0.24; 95% CI: -1.50 – 1.01; P = 0.71; Fig. 3A] between Amantadine and placebo groups in terms of functional improvement. There was a huge amount of heterogeneity (i^2^ = 92%; P = 0.0003; Fig. 3A) and the 95% CI touched the line of null effect.

On the other hand, the pooled SMD via fixed-effect model showed a significant difference [SMD: -0.61; 95% CI: -0.88 to −0.34; P < 0.0001; Fig. 3B] on functional improvement which favored Amantadine group against placebo group.

Taken together, results from the random-effects analysis, as presented in Fig. 3A, and from the fixed-effect analysis in Fig. 3B, suggested that treatment favored Amantadine and may have an effect on functional improvement among moderate-severe TBI patients.

#### 3.1.6 Sensitivity Analysis

One triple-blind placebo-controlled trial by Shimia and colleagues (Shimia et al., 2021) used Amantadine versus placebo at the advent of their study (Giacino et al., 2012). However, full-text article was not retrievable, hence, in keeping with the methods of the current study a priori, the study of Shimia and colleagues (Shimia et al., 2021) was not synthesized in the primary analysis - restricted to full-text studies on Amantadine compared to placebo and at low or unclear risk of bias - but were included in the sensitivity analyses for the outcomes related to functional improvement to ascertain whether their data overestimated or changed the direction of effect estimates.

**Fig. 4:**
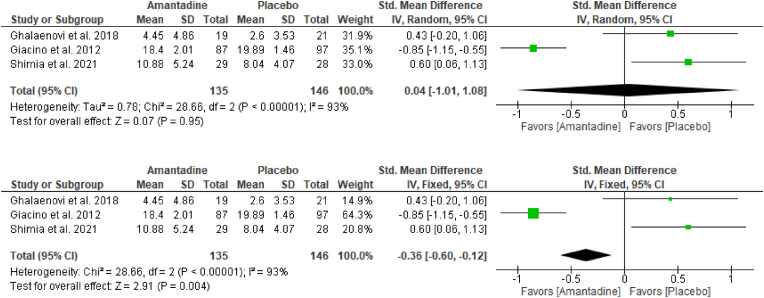
Forrest plot via random-effects model (Fig. 4A, top) and fixed-effect model (Fig. 4B, bottom) of Amantadine versus placebo on functional improvement among moderate-severe TBI patients (Sensitivity Analysis).

The sensitivity analysis of the effect of Amantadine in Fig. 4A and B, including the data from the published abstract of Shimia and colleagues, (Shimia et al., 2021) still pointed to the same direction, and had not overestimated the statistical estimates to a large extent in terms of functional improvement as aligned with the findings of the primary analyses in Fig. 3A and B, respectively.

**Fig. 5:**
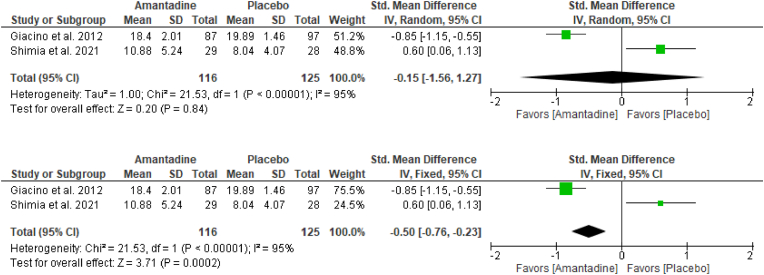
Forrest plot via random-effects model (Fig. 5A, top) and fixed-effect model (Fig. 5B, bottom) of Amantadine versus placebo on functional improvement among severe TBI patients (Sensitivity Analysis).

Also, given the limitation of DRS for the assessment of moderate TBI cases, sensitivity analysis was restricted to adult severe TBI patients in Fig. 5A and B – that is, taking out the composite data of moderate-severe TBI patients obtained from the study of Ghalaenovi and colleagues (Ghalaenovi et al., 2018) – showed no changes in the direction of results and statistical estimates as seen in the primary analyses in Fig. 3A and B, respectively.

Thus, sensitivity analyses confirmed that the primary analysis on the effect of Amantadine versus placebo on functional improvement among moderate-severe TBI patients is robust.

#### 3.1.7 Trial sequential analysis

TSA was used to calculate the RIS to demonstrate or reject a decrease of 1.37 in mean DRS scores among the Amantadine group, as observed in the primary analysis. Model variance-based heterogeneity-adjustment is 92% obtained from inconsistency index. Finally, the RIS = 750 moderate-severe TBI participants, which was not exceeded by the total number of observed TBI patients in the primary analysis (n = 224).

**Fig. 6:**
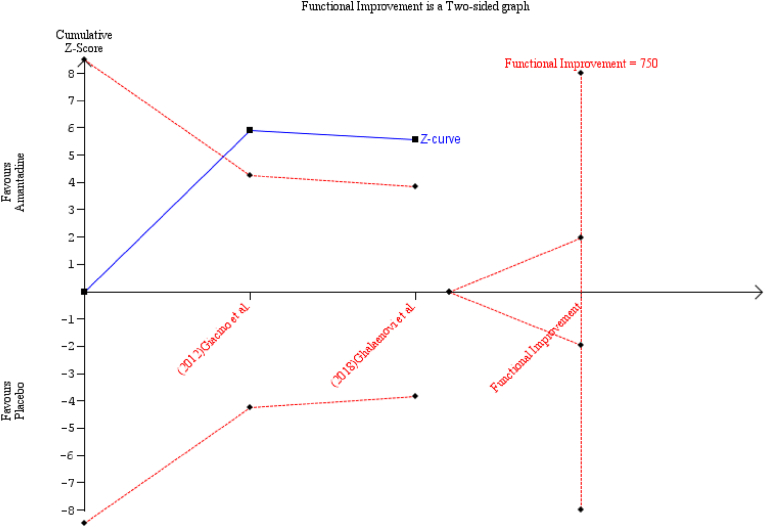
Trial sequential analysis plot with parameter estimates of α = 0.05, β = 0.2, MD = -1.37, and heterogeneity-adjustment = 92%.

In the TSA plots, the red dashed lines in Fig. 6 represent the trial sequential monitoring and futility boundaries using the O'Brien-Fleming α-spending and β-spending functions, respectively. The solid blue line is the cumulative Z-curve and represents the accumulating data each time a study is published surrounding the clinical foregrounding question of the current study. In the same figure, the Z-curve crossed the boundary for statistical significance (Z = 1.96) and the O'Brien-Fleming superiority boundary, but did not cross the RIS, thereby suggesting that with n = 224, evidence has enough power to conclude that Amantadine is still superior than placebo at the conduct of the current study, however, more studies with high validity must still be performed with the goal of reaching the RIS = 750 qualified patients to establish the confidence in the recommendation and use of Amantadine among moderate-severe TBI adult patients.

## 4 Discussion

### 4.1 Summary of main results

Three studies are evaluated with a total of 281 adult TBI patients meeting the inclusion criteria of this review. High certainty evidence suggests that Amantadine has beneficial effect on functional improvement and demonstrates superiority compared to placebo. However, observed participants are still few to achieve recommendation for the use of Amantadine among adult patients with moderate-severe TBI.

### 4.2 Overall completeness and applicability of evidence

The studies are conducted across different clinical sites and critical care units and hospitals in the USA and Iran. Eligible injury patients after trauma were assessed and were followed-up from 2 weeks to 24 weeks. Moreover, the findings of this review are transferable not only for severe cases but to moderate TBI patients and accordingly applicable when DRS scores are used as endpoints of functional improvement in clinical settings. Pediatric population is still scarcely untouched in terms of outcomes related to DRS to become feasible for meta-analytic studies, hence, adult trauma patients are the focus of the current study. However, the current study could not absolutely establish that earlier treatment seemed to improve patients more rapidly than later treatment while they were on Amantadine as the timing of treatment and follow-up periods are widely differentiated across studies, hence, subgroup analysis could not be performed. Furthermore, since studies introduced Amantadine in the eligible participants in varying timepoints and duration after trauma, the included studies reported overlapping time-to-event analyses. Also, based on the eligible study pool, Amantadine is superior than placebo, however, observed adult TBI patients are still lacking in the required numbers for the use Amantadine for functional improvement in terms of DRS scores, hence, careful assessments during admission and high-quality clinical trials should still be performed to further the results of the current study. Moreover, subgroup analyses were not performed due to the low number of studies to pool such subgroups as potential effect modifiers.

### 4.3 Quality of the evidence

There was a high certainty of evidence for functional improvement in terms of DRS scores, mainly because the effect estimates derived from the pooled studies possess low to unclear risks of bias, are directly applicable to intended trauma patients and could potentially demonstrate dose-gradient effects as implied on the Amantadine regimen employed in each study.

### 4.4 Potential biases in the review process

The methods of this review are designed to minimize additional bias. Relevant studies are searched to summarize the best available evidence during the conduct of this review. Data search found several case reports and retrospective cohort studies, but a paucity of randomized placebo-controlled clinical trials. Furthermore, the published studies answering the foreground question were found to be heterogenous in design and difficult to compare, hence, a low number of included studies subjected to analyses. Additional sensitivity analyses, TSA and assessment of evidence certainty were carried out to strengthen the findings of the current study. However, there are possible indications of publication bias from the universe of studies reporting DRS scores as functional improvement, surrounding the clinical foreground question. Conventionally, visual inspection of the funnel plots is not conclusive when there are less than 10 studies included in the synthesis, therefore, not presented on this review, in so far as that determining such bias using formal statistical analysis may otherwise possess low power. Studies that did not meet the inclusion criteria were also restricted in the primary analysis to avoid overestimating the effect of Amantadine.

### 4.5 Agreements and disagreements with other studies or reviews

The current review provides promising evidence of Amantadine for functional improvement among adult patients with moderate to severe TBI, however, the findings illustrate a still-limited body of knowledge in agreement with previous review published by Loggini and colleagues (Loggini et al., 2020) in 2020 for the role of Amantadine in cognitive recovery after TBI.

The timing of Amantadine administration shows conflicting results, thus, employing meta-analysis and sequential analysis are relevant. The work of Giacino and colleagues (Giacino et al., 2012) favors Amantadine during the first 4 weeks of treatment as also shown by the results of a meta-analysis conducted by Mohamed and colleagues (Mohamed et al., 2022) wherein better effects are achieved when Amantadine is administered for less than 1 month. However, opposing evidence is published with the result produced by Ghalaenovi and colleagues (Ghalaenovi et al., 2018) showing no significant relation of DRS score improvement as compared to placebo regardless of the timing of administration. Meythaler and colleagues (Meythaler et al., 2002) posit a significant improvement in DRS in 35 patients initially treated with Amantadine during the first 4–6 weeks in their double-blind, randomized, placebo-controlled cross-over study. Since the timing of treatment and follow-up periods are heterogeneous across the included studies of the current review, it could not differentiate the effect of earlier treatment than later treatment. Overall, Meythaler and colleagues (Meythaler et al., 2002) demonstrates improvement of DRS when patients are treated with Amantadine versus placebo; Giacino and colleagues (Giacino et al., 2012) demonstrates faster improvement rate of DRS, while Ghalaenovi and colleagues (Giacino et al., 2012) shows no benefit in the use of Amantadine. It is possible that the challenge of achieving homogeneous results in terms of timing of administration is brought by the inclusion of TBI patients with different mechanisms of injury to the presently available body of knowledge concerning the use of Amantadine for TBI. In fact, research suggests that the mechanism of injury may play a role in determining the neurochemical factors which links to the pathophysiology associated with TBI recovery (Toide, 1990). Furthermore, with the growing evidence of functional improvement beyond 6 months after injury it would be worth considering to conduct studies that follow up patients over 1 year in order to gauge the longer-term effects on disability and impairment, and the steadiness in functional recovery in patients treated with Amantadine (Bareham et al., 2019; Wilkins et al., 2019). Moreover, outcome measures should be more comprehensive, standardized, and should not possess limitations in evaluating functional improvement.

## 5 Conclusion

Evidence of this review show that the use of Amantadine may have a beneficial effect on functional outcome in moderate to severe traumatic brain injuries among adult patients. Given the still-limited body of knowledge, more relevant studies must be made exploring the impact of Amantadine therapies on promoting functional recovery within the brain injury rehabilitation care continuum, with the goals of achieving larger sample sizes and establishing the early- or later-treatment beneficial effects.

## Disclosures

The authors have no competing interest to disclose.

## Funding statement

There was no funding provided for this research.

## Previous presentations (if any)

None.

## Credit author statement

Hantz Siy: Conceptualization, Methodology, Investigation, Data Curation, Writing – Original Draft, Writing – Review & Editing, Visualization. Michael Gimenez: Conceptualization, Investigation, Writing – Review & Editing, Supervision. Wesley Rosete: Methodology, Writing – Review.

## Declaration of competing interest

The authors have no competing interest to disclose.
