# Supplementary material for: Amantadine for functional improvement in patients with traumatic brain injury: A systematic review with meta-analysis and trial sequential analysis
Source: Brain Spine. 2024 Feb 23;4:102773. doi: 10.1016/j.bas.2024.102773 (PMC10924175; doi:10.1016/j.bas.2024.102773)
Supplement: Multimedia component 1 [file mmc1.docx]

| **Amantadine compared to placebo for TBI** | | | | | | | | | | | |
| --- | --- | --- | --- | --- | --- | --- | --- | --- | --- | --- | --- |
| **Certainty assessment** | | | | | | | **Summary of findings** | | | | |
| **Participants (studies) Follow-up** | **Risk of bias** | **Inconsistency** | **Indirectness** | **Imprecision** | **Publication bias** | **Overall certainty of evidence** | **Study event rates (%)** | | **Relative effect (95% CI)** | **Anticipated absolute effects** | |
|  |  |  |  |  |  |  | **With Placebo** | **With Amantadine** |  | **Risk with Placebo** | **Risk difference with Amantadine** |
| **Functional improvement (follow-up: range 2 weeks to 24 weeks)** | | | | | | | | | | | |
| 224 (2 RCTs) | not serious | serious ^a^ | not serious | serious ^b^ | publication bias strongly suspected ^c^ strong association all plausible residual confounding would reduce the demonstrated effect dose response gradient | ⨁⨁⨁⨁ High | 118 | 106 | - | - | SMD **0.61 lower** (0.88 lower to 0.34 lower) |

**CI:** confidence interval; **SMD:** standardized mean difference

#### Explanations

a. Effect estimates from both studies have small to large effect sizes, do not point to the same direction, with 95%CI that are not overlapping. Heterogeneity: i² > 50% (P < 0.05)
b. n is less than RIS with one study effect estimate touching the line of null effect
c. Presence of funnel plot asymmetry. However, the included studies for this outcome are less than 10. Funnel plot asymmetry has a low power to determine publication bias from an outcome with studies less than 10
