# Supplementary material for: Amantadine for functional improvement in patients with traumatic brain injury: A systematic review with meta-analysis and trial sequential analysis
Source: Brain Spine. 2024 Feb 23;4:102773. doi: 10.1016/j.bas.2024.102773 (PMC10924175; doi:10.1016/j.bas.2024.102773)
Supplement: Multimedia component 2 [file mmc2.docx]

| **Summary of findings:** | | | | | | |
| --- | --- | --- | --- | --- | --- | --- |
| **Amantadine compared to Placebo for TBI on DRS** | | | | | | |
| **Patient or population:** adult patients with moderate-severe TBI **Setting:** Clinical sites and critical care units and hospitals in the USA and Iran **Intervention and Comparison:** Amantadine versus placebo | | | | | | |
| Outcome № of participants (studies) | Relative effect (95% CI) | **Anticipated absolute effects (95% CI)** | | | Certainty | What happens |
|  |  | Without Amantadine | With Amantadine | **Difference** |  |  |
| Functional improvement follow-up: range 2 weeks to 24 weeks № of participants: 224 (2 RCTs) | - | Mean functional improvement ranged from 2.6 (SD = 3.53) to 19.89 (SD = 1.45) | Mean functional improvement ranged from 4.45 (SD = 4.86) to 18.4 (SD = 2.01) | SMD **0.61 lower** (0.88 lower to 0.34 lower) | ⨁⨁⨁⨁ High ^a, b, c^ | Amantadine improves DRS scores as a measure of functional improvement in TBI patients |
| **CI:** confidence interval; **SMD:** standardized mean difference | | | | | | |
| **GRADE Working Group grades of evidence** **High certainty:** we are very confident that the true effect lies close to that of the estimate of the effect. **Moderate certainty:** we are moderately confident in the effect estimate: the true effect is likely to be close to the estimate of the effect, but there is a possibility that it is substantially different. **Low certainty:** our confidence in the effect estimate is limited: the true effect may be substantially different from the estimate of the effect. **Very low certainty:** we have very little confidence in the effect estimate: the true effect is likely to be substantially different from the estimate of effect. | | | | | | |

#### Explanations

a. Effect estimates from both studies have small to large effect sizes, do not point to the same direction, with 95%CI that are not overlapping. Heterogeneity: i² > 50% (P < 0.05)
b. n is less than RIS with one study effect estimate touching the line of null effect
c. Presence of funnel plot asymmetry. However, the included studies for this outcome are less than 10. Funnel plot asymmetry has a low power to determine publication bias from an outcome with studies less than 10
